# Dissecting Wounds

**Published:** 1860-07

**Authors:** 


*Dissecting Wounds.*—M. Ch. Phillips, a surgeon well known in Paris by very successful labors in urinary pathology and practice, has been in a very precarious state from a dissecting wound, but is now recovering.

